# Identification of Distinct Immune Subtypes in Colorectal Cancer Based on the Stromal Compartment

**DOI:** 10.3389/fonc.2019.01497

**Published:** 2020-01-10

**Authors:** Rongfang Shen, Ping Li, Bing Li, Botao Zhang, Lin Feng, Shujun Cheng

**Affiliations:** ^1^State Key Laboratory of Molecular Oncology, Department of Etiology and Carcinogenesis, National Cancer Center/National Clinical Research Center for Cancer/Cancer Hospital, Chinese Academy of Medical Sciences and Peking Union Medical College, Beijing, China; ^2^Beijing Key Laboratory of Pediatric Hematology Oncology, National Key Discipline of Pediatrics (Capital Medical University), Key Laboratory of Major Diseases in Children, Ministry of Education, Hematology Oncology Center, Beijing Children's Hospital, Capital Medical University, National Center for Children's Health, Beijing, China; ^3^Department of Peritoneal Cancer Surgery, Beijing Shijitan Hospital, Capital Medical University, Beijing, China; ^4^Department of Neuro-oncology, Neurosurgery Center, Beijing Tiantan Hospital, Capital Medical University, Beijing, China

**Keywords:** microdissection, colorectal cancer, tumor environment, weighted gene coexpression network analysis, immune subtypes, immunotherapy

## Abstract

The tumor environment is of vital importance for the incidence and development of colorectal cancer. Increasing evidence in recent years has elaborated the vital role of the tumor environment in cancer subtype classification and patient prognosis, but a comprehensive understanding of the colorectal tumor environment that is purely dependent on the stromal compartment is lacking. To decipher the tumor environment in colorectal cancer and explore the role of its immune context in cancer classification, we performed a gene expression microarray on the stromal compartment of colorectal cancer and adjacent normal tissues. Through the integrated analysis of our data with public gene expression microarray data of stromal and epithelial colorectal cancer tissues processed through laser capture microdissection, we identified four highly connected gene modules representing the biological features of four tissue compartments by applying a weighted gene coexpression network analysis algorithm and classified colorectal cancers into three immune subtypes by adopting a nearest template prediction algorithm. A systematic analysis of the four identified modules mainly reflected the close interplay between the biological changes of intrinsic and extrinsic characteristics at the initiation of colorectal cancer. Colorectal cancers were stratified into three immune subtypes based on gene templates identified from representative gene modules of the stromal compartment: active immune, active stroma, and mixed type. These immune subtypes differed by the immune cell infiltration pattern, expression of immune checkpoint inhibitors, mutation landscape, extent of mutation burden, extent of copy number burden, prognosis and chemotherapeutic sensitivity. Further analysis indicated that activation of the *NF-kB* signaling pathway was the major mechanism causing the no immune infiltration milieu in the active stroma subtype and that inhibitors of the *NF-kB* signaling pathway could be candidate drugs for treating patients with an active stroma. Overall, these results suggest that characterizing colorectal cancer by the tumor environment is of vital importance in predicting patients' clinical outcomes and helping guide precision and personalized treatment.

## Introduction

Colorectal cancer is the third most common cancer and ranks second in terms of cancer-related mortality (1). Most colorectal cancer patients die because of a late diagnosis, recurrence after surgical excision, or resistance to chemotherapy or radiotherapy. Patients with the same American Joint Committee on Cancer (AJCC) stage and pathomorphological features are given consistent treatment regimens and often have distinct prognoses and treatment responses. The current treatment dilemma underscores the critical need to improve colorectal cancer classification with distinct molecular features and survival outcomes for reasonable clinical treatment decisions.

The tumor epithelium and surrounding microenvironment closely interact through the extracellular matrix or secreted factors such as exosomes, cytokines, and angiogenic factors (2). The depiction of a transcriptome map of the altered biological processes in the epithelial and stromal compartments will not only allow investigators to comprehensively understand the mechanism of cancer initiation and the complex coevolving relationships between the intrinsic and extrinsic factors of tumors (3) but also help in the detection of druggable epithelial–stromal crosstalk targets (4). Nishida previously used a laser capture microdissection (LCM)-processed miRNA and gene expression microarray to reveal two miRNA clusters with high expression in the cancer stroma (5). However, the major changes in the biological features of the epithelial and stromal compartments between colorectal cancer and adjacent normal tissues remain poorly understood. A systematic analysis of the different compartments of colorectal tissues is needed to better understand the mechanisms of tumor initiation.

The tumor microenvironment (TME), which includes blood vessels, lymph vessels, immune cells and mesenchymal cells, is a complex ecosystem of stromal cells and plays a critical role during tumorigenesis and progression. Previously identified transcriptome subtypes of colorectal cancer associated with a poor prognosis, including the stem (6), serrated (7), and mesenchymal (8) subtypes, are enriched with genes derived from the activated stromal compartment. The differentially expressed genes of preoperative chemoradiotherapy-treated rectal carcinomas between responders and non-responders are mainly contributed by the stroma and not tumor glands (9). The TME is a major contributing factor for patient outcomes and chemoradiotherapy treatment responses (10, 11). Additionally, recent studies have indicated that TME characteristics are closely associated with the response to immune checkpoint blockade (ICB) treatment (12, 13). For example, epithelial-mesenchymal transition (EMT)-, stroma- and angiogenesis-related signatures are significant contributors to ICB treatment resistance (14, 15), while the high infiltration of cytotoxic T cells can elicit an effective immune response to attack tumor cells (16). Thus, the surrounding tumor environment can shape the biological behavior and the reaction of tumor cells to a drug regimen.

Despite increasing evidence proving the crucial role of the immune context in determining immunological treatment reactions and prognoses, most studies have focused on bulk tumor transcriptomes, with mixed data from the tumor epithelium and stroma. Few studies have focused on the changes in immunological responses purely modified by the surrounding immune context. In this study, we first described the major biological process changes in the epithelium and stroma of colorectal cancers and adjacent normal tissues and defined three diverse colorectal immune subtypes, namely, the “active immune,” “active stroma” and “mixed type” subtypes, based on the top 40 most connected genes from the identified network module by adopting weighted gene coexpression network analysis (WGCNA). These subtypes had distinct immune environments, genomic contexts, and ICB treatment and chemotherapy response tendencies. Drugs targeting the *NF-kB* signaling pathway could convert cold tumors into hot tumors. Overall, this work proposes a new colorectal cancer classification system that is purely based on the tumor environment and has the potential to guide treatment decisions.

## Materials and Methods

### Clinical Samples

Tissues from six patients with colorectal cancer and 6 adjacent normal tissue samples (located more than 5 cm away from the tumor edge) were obtained during surgery. Four patients had paired tumor and adjacent normal samples, and the other four samples were obtained from distinct patients. The obtained tissues were independently morphologically reviewed by two experienced pathologists to confirm the diagnostic accuracy. Representative histopathological images are showed in Figure S1. Fresh tissues were cleaned with normal saline solution and frozen at -80°C within 30 min before RNA preparation. No chemotherapy or radiotherapy was administered to the patients prior to therapeutic resection. All patients underwent resection of the primary tumor at the Department of Peritoneal Cancer Surgery, Beijing Shijitan Hospital, Capital Medical University (Beijing, China) between February 2016 and December 2016. Written informed consent was obtained from all patients, and the study protocol was approved by the Ethics Committee of the National Cancer Center/Cancer Hospital, Chinese Academy of Medical Sciences and Peking Union Medical College.

### Microdissection Processing of Colorectal Tissue and Gene Expression Microarray

The stromal compartment of the cancer and normal samples was obtained manually by microdissection. All colorectal tissues were embedded in OCT (Thermo Fisher) and cut into 10-μm slices with a freezing microtome. The frozen tissue slices were then placed in hematoxylin for 2-5 s. Next, stromal tissues were isolated with a needle under a microscope and collected in Eppendorf tubes. Schematic diagram of the marked stromal compartment processed through microdissection are illustrated in Figure S1. Total RNA extracted from the stromal compartment of the cancer and normal samples was labeled and hybridized to Agilent 8^*^60K Whole Human Genome Oligo Microarrays (G4851B) according to the manufacturer's protocol. All RNA integrity numbers (RINs) of the microdissected sample compartments were greater than 7.0, and RNA integrity was assessed using an Agilent 2100 Bioanalyzer (Agilent Technologies, Santa Clara, CA, USA). The raw and processed data are publicly available at the Gene Expression Omnibus (GEO) website under accession number GSE136735. A previously published dataset, GSE35602, which contains epithelial and stromal regions of colorectal cancer and normal tissues through LCM, was integrated to identify specific modules of different colorectal compartments (5). The background correction and normalization of raw data were processed by the R package “limma.” The Combat algorithm was used to eliminate technological bias caused by different microarray platforms between the two datasets described above (17). Since the stromal compartment in our microarray profile was obtained manually by microdissection and the GSE35602 dataset was processed through LCM, we used the term “microdissection” to generalize the two methods in our integrated data in this study. Basic clinical characteristics of the enrolled samples in the two datasets are demonstrated in Table S1.

### Public Colorectal Cancer Transcriptional Profiles

In this study, we used the GSE39582 dataset, which is the largest microarray cohort with complete survival information among published colorectal cancer expression spectra, and the TCGA-COADREAD (TCGA-COAD and TCGA-READ) cohort. The R packages “GEOquery” (18) and “TCGAbiolinks” (19) were implemented to download the processed expression matrix and clinical data of the GEO datasets and the TCGA-COADREAD cohort, respectively, in March 2018. Available TCGA “level 3” gene expression data of the TCGA-COADREAD cohort were downloaded. Log2 (transcripts per kilobase million (TPM) + 1)-transformed normalized values were applied for immune cell infiltration pattern estimation and SubMap analysis. The survival information data of the TCGA cohort, including overall survival and relapse-free survival, were downloaded from the UCSC Xena browser, while other clinical data, such as age, sex, and microsatellite information, were obtained by the R package “TCGAbiolinks.” For genes with multiple probe sets, the mean expression levels were used as the gene expression values.

### ICB Cohorts

Four pretreatment tumor expression profiles of ICB cohorts were included in this study to assess the similarity between the identified subtypes and the ICB treatment response. The data of melanoma patients treated with anti-PD-1 (accession number: GSE78220) (15), metastatic melanoma patients treated with *MAGE-A3* immunotherapy (accession number: GSE35640) (20), and mice AB1-HA tumors treated with anti-*CTLA-4* (accession number: GSE63557) (21) were obtained from the National Center for Biotechnology Information (NCBI) GEO database (http://www.ncbi.nlm.nih.gov/geo/). The data of patients with metastatic urothelial tumors from the IMvigor210 cohort (22) treated with anti-PD-L1 were obtained from http://research-pub.gene.com/IMvigor210CoreBiologies/. Processed fragments per kilobase million (FPKM) data of the GSE78220 cohort were transferred into TPM data. Gene expression in the IMvigor210 cohort was normalized by implementing the “voom” function in the “limma” package. The processed normalized data of the remaining two microarray cohorts were obtained by the “GEOquery” package.

### Identification of Representative Modules of Colorectal Compartments

To illustrate the biological changes in the epithelial and stromal compartments between colorectal cancer and normal tissues, we applied WGCNA to identify the representative transcriptional network modules of the different compartments. Genes with a low dynamic range were excluded, and only the top 8000 genes with the highest standard deviation were evaluated to construct the coexpression network. The freely available statistical analysis software (“WGCNA” R package) and R tutorials for constructing the weighted gene coexpression network have been described previously (23).

### Identification of Colorectal Cancer Subtypes Based on Microenvironment Features

To clarify the impact of the surrounding environment of the tumor on colorectal cancer, nearest template prediction (NTP) (24) was applied to assign patients into three transcriptomic subtypes. NTP performed class prediction using predefined gene markers and returned the significance level of each sample prediction with a nominal *P*-value. We set the Benjamini-Hochberg (BH)-corrected false discovery rate (FDR) to 0.2 as the prediction threshold for the significant classification of a sample according to a previous report (6). The tumor purity information of the TCGA-COADREAD and GSE39582 cohorts was extracted from previous TCGA research (25) using the ABSOLUTE method (26) and estimated using the R package “estimate” (27).

### Subclass Mapping

The Subclass Mapping (SubMap) method (28) was used to evaluate the similarity between the identified subtypes and the immunotherapy-treated patients. The SubMap algorithm uses the Gene Set Enrichment Analysis (GSEA) function to evaluate the extent of commonality of the different subtypes in independent datasets. *P*-values were used to evaluate the similarity, and the lower the *P*-values were, the higher the similarity. Recommended default parameters, including the number of marker genes (100), random permutations for the enrichment score (100), and random permutations for Fisher's statistics (1000), were used. The R package “complexHeatmap” (29) was implemented to visualize the results of the SubMap analysis.

### Correlation of TME-Based Subtypes With Mutations and Copy Number Aberrations

Significantly mutated genes were generated by MutSigCV_1.41 for the TCGA-COADREAD cohort accessed from the mutation annotation file (https://gdc.cancer.gov/about-data/publications/panimmune).

MutSigCV (30) identifies significantly mutated genes more than expected by chance. The tumor mutation burden (TMB) of each patient was calculated as the total number of non-synonymous mutations per megabase. Fisher's exact test was applied to detect different mutated genes between the active immune and active stroma compartments. For copy number analysis, we applied GISTIC_2.0 to identify significantly amplified or deleted genomes (31). The burden of copy number loss or gain was calculated as the total number of genes with copy number changes at the focal and arm levels. NTP, SubMap, MutSigCV_1.41 and GISTIC_2.0 are freely available on GenePattern (https://cloud.genepattern.org).

### Functional Analysis and GSEA

The R package “clusterProfiler” (32) was applied for the Gene Ontology (GO) analysis of four core transcriptional modules inferred from the WGCNA. GSEA was applied to enrich hallmark gene sets downloaded from the Molecular Signatures Database (MSigDB). Input genes were ranked in descending order according to the log2FC values. Enrichment significance was estimated using default settings and 1000 permutations. Benjamini–Hochberg-adjusted *P*-values less than 0.05 were considered significantly enriched.

### Quantifying Tumor-Infiltrating Immune Cells

To estimate the immune and stromal cell infiltration patterns in colorectal cancer, the Microenvironment Cell Populations-counter (MCP-counter) method (33) using colorectal cancer gene expression profiles was applied. MCP-counter is a robust and highly informative method that quantifies eight types of immune cells and two types of stromal cells based on marker genes.

### Connectivity Map Analysis

To further illustrate the molecular mechanism underlying the difference in immunogenicity between active immune and active stroma compartments and identify potentially useful drugs, we performed connectivity map analysis (34) using the 150 genes with the most significant fold changes (up- and downregulated). In total, we submitted 300 genes to the CMap website (https://clue.io/). All 300 genes were significantly different under the criterion of FDR-adjusted *P* < 0.05 in the TCGA-COADREAD cohort.

### Predicting the Clinical Chemotherapeutic Response

The R package “pRRophetic” (35)was applied to estimate the chemotherapeutic response of 5-fluorouracil and cisplatin in the TCGA-COADREAD and GSE39582 cohort. Cell lines originating from the digestive system and the “cgp2016” dataset were applied when implementing the “pRRopheticPredict” function. This methodology fitted the ridge regression model based on baseline gene expression and drug sensitivity of the cell line, thus allowing the prediction of the clinical chemotherapeutic response using only patients' baseline gene expression data (36). Drug sensitivity was measured by the concentration required for 50% of cellular growth inhibition (IC50).

### Statistical Analysis

All statistical analyses were performed using R (https://www.r-project.org/). The Wilcoxon rank-sum test was adopted to compare differences between two groups. We used the Kruskal–Wallis test to evaluate significant differences when comparing more than two groups. The “edgeR” pipeline was adopted for the differential expression analysis. Survival probabilities were estimated with the Kaplan-Meier method, and the log-rank (Mantel-Cox) test was used to compare the survival distributions between two groups. A log-rank *P* < 0.05 was considered statistically significant.

## Results

### Transcriptional Map of the Epithelium and Stroma in Colorectal Cancer and Adjacent Normal Tissues

**Figure 1:**
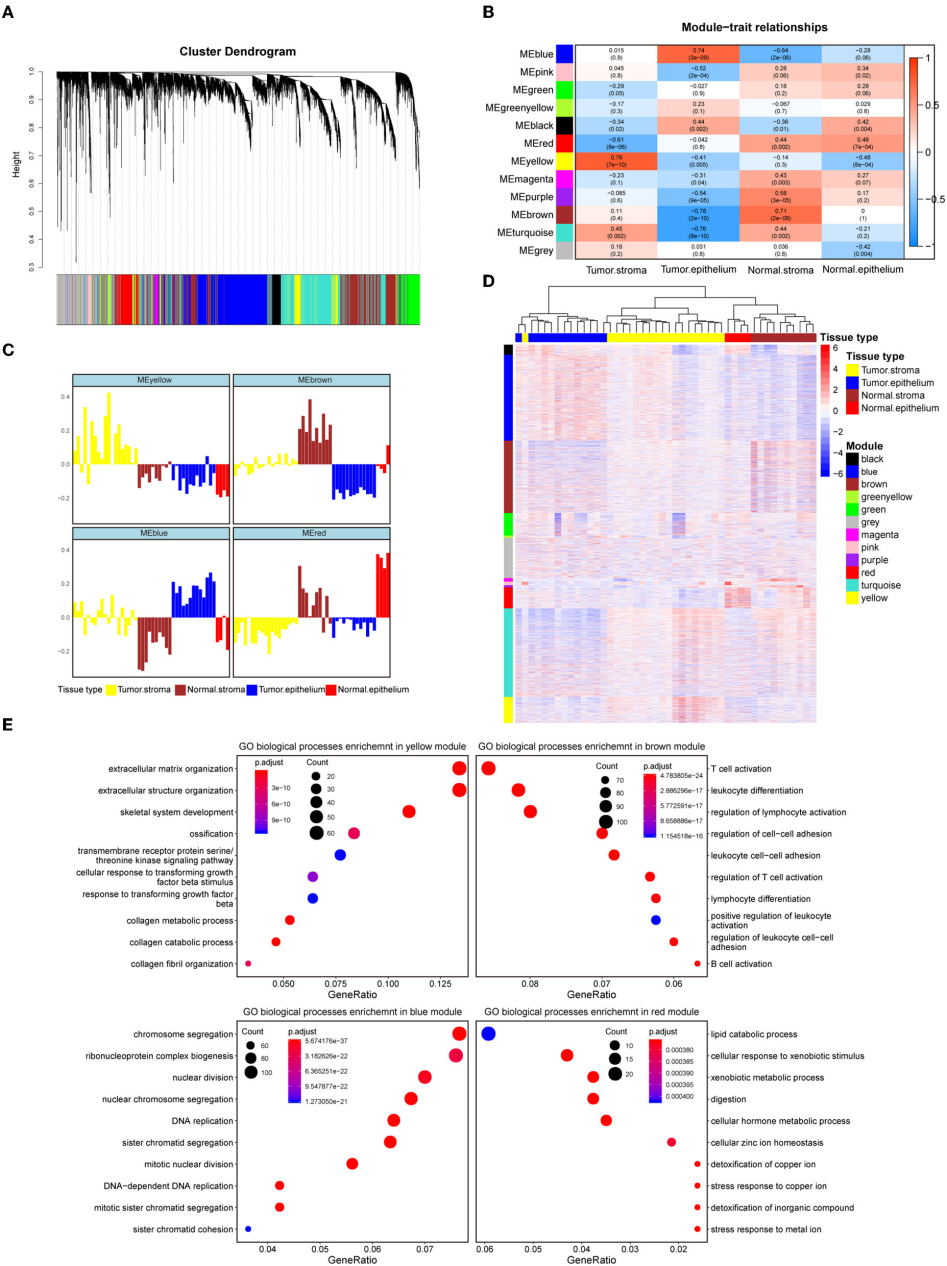
Identification of modules representative of diverse colorectal compartments. **(A)** Hierarchical cluster dendrogram of the top 8000 genes with the highest standard deviation. The identified modules underneath the tree are color coded. **(B)** Heat map of module-trait associations; rows represent the module eigengene, and columns represent clinical traits. The Spearman correlation and significance level enclosed in brackets are labeled in each cell. The color intensity of the cell corresponds to the correlation coefficient. **(C)** Eigengene bar plot of the yellow, brown, blue and red modules. Samples are ordered by the tumor stroma, normal stroma, tumor epithelium, and normal epithelium and are labeled in yellow, brown, blue and red, respectively. The module eigengene is defined as the first principal component of the module's expression matrix. **(D)** Hierarchical clustering heat map of the top 8000 genes with the highest standard deviation. Genes are ordered by the modules, ranging from the black module to the yellow module. The samples' corresponding tissue compartments are annotated in the column annotation panel on the top side of the heat map. The color intensity indicates the relative expression level of the genes. **(E)** Dot plot of the biological process enrichment results. The top 10 GO terms with the highest enrichment levels are shown; the dot size and color represent the gene count and enrichment level, respectively.

To systematically characterize the expression patterns of the epithelial and stromal compartments during colorectal carcinogenesis, we performed WGCNA on the 8000 most variable genes (Table S2) and identified 12 transcriptional modules with gene numbers ranging from 47 to 1874 genes (Figure 1A). In relating these modules to tissue compartment information by correlating the eigengenes of each module with compartment traits, four modules with the most significant correlations to the tumor epithelium (blue module), tumor stroma (yellow module), normal epithelium (red module), and normal stroma (brown module) were identified (Figure 1B). The eigenvalue of these selected modules was the highest within the most closely related samples relative to the samples in the remaining three groups, which also confirmed their representation (Figure 1C). The heat map in Figure 1D shows the expression levels of all modules, and these four modules have remarkably high expression levels with their most correlated samples. Given the representativeness of these four modules, biological process enrichment analysis was applied to investigate the related properties of tissue glands and the stroma in the process of tumorigenesis (Figure 1E, Table S3). The yellow module, which had a markedly high expression level in the colorectal tumor stroma, was characterized by the overexpression of genes involved in extracellular matrix organization, the cellular response to transforming growth factor-β stimulus and the collagen metabolic process. Immune-related pathways such as T cell activation, B cell activation and lymphocyte differentiation were enriched in the brown module, whose expression level was the highest in the normal stroma. Different GO biological processes between the normal stroma and tumor stroma in the colorectum consisted of the transformation of immune-infiltrating cells (from B lineage cells and T cells to fibroblasts) (Figures S2A,B). Cancer-associated fibroblast-secreted cytokines such as IL-6 influence the phenotype of neoplastic cells, including proliferation, migration, and angiogenesis (37). Consistent with this finding, the overexpression of *CDK1, EREG* and *ANLN* along with several biological processes related to cell proliferation, such as nuclear division, DNA replication and regulation of the mitotic cell cycle phase transition, were enriched in the blue module. The red module was characterized by genes involved in metabolic pathways, including lipid catabolic processes and steroid metabolic processes (Figure 1E). The systematic analysis of stromal and epithelial tissues between colorectal cancer and adjacent normal tissues indicated that the tumor stroma might provide a suitable niche prompting tumor cell proliferation and invasion, while a normal environment with abundant immune cells helps maintain the function of the normal colorectum.

### Identification of Colorectal Transcriptomic Subtypes Based on the Surrounding Microenvironment

**Figure 2:**
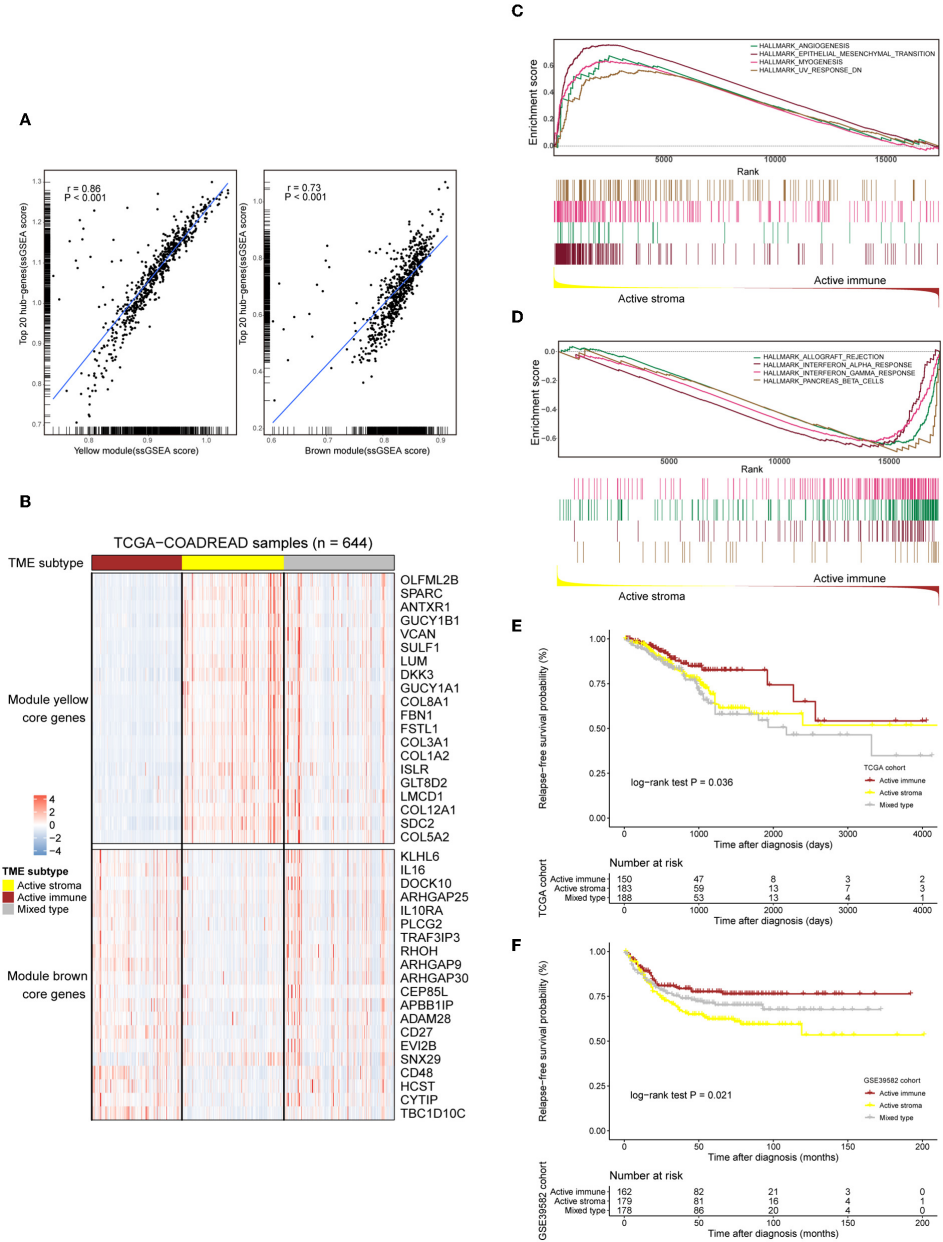
Selected gene template-based NTP reveals subtypes characterized by a distinct immune context and is associated with survival outcomes. **(A)** Correlation scatter plot of the 20 selected hub genes and genes in the whole module. The enrichment score of each sample in a given gene set was calculated through the ssGSEA algorithm. The correlation coefficient and the significance level of the test are annotated at the top left of the figure. Left: yellow module. Right: brown module. **(B)** Heat map showing the expression patterns of the selected hub genes in the TCGA-COADREAD cohort based on the identified immune subtypes. Genes annotated on the right side of the heat map are the selected core genes separated by the module to which they belong. The immune subtype classification of each sample is annotated at the top side of the heat map. Color intensities indicate the expression level of the genes. **(C,D)** GSEA plot of the enriched hallmark gene sets derived from the Molecular Signatures Database (MSigDB). The running score and preranked list are placed at the top and bottom of the GSEA plot. The middle indicates whether members of gene sets appear in the ranked list of genes. Multiple gene set enrichment results indicated by corresponding colors are shown on the same figure. **(E,F)** Kaplan–Meier curves for relapse-free survival (RFS) of the three identified immune subtypes in the TCGA-COADREAD and GSE39582 cohorts.

The TME plays a crucial role in colorectal tumorigenesis and progression. Considering the vital importance of the tumor environment, we classified colorectal cancer patients into distinct tumor types based on the context of the tumor environment for further analysis. The top 20 genes with the highest network degree in the yellow module and brown module were selected as the gene templates (Table S4). The gene templates and genes in the whole module were highly correlated in both the yellow (Spearman's correlation coefficient = 0.86, *P* < 0.001, Figure 2A, left) and brown (Spearman's correlation coefficient = 0.73, *P* < 0.001, Figure 2A, right) modules. We assigned patients into three immune subtypes, namely, the “active stroma,” “active immune” and “mixed type” subtypes by applying NTP analysis using these curated gene templates (Figure 2B). NTP, a well-acknowledged signature-based disease classification method, uses only a list of gene signatures to assess the possibility of each single sample belonging to a specific classification. As shown in Figure 2B, 33.7% (217/644) of the colorectal cancer patients from the TCGA-COADREAD cohort were predicted as having an active stroma compartment, 29.8% (192/644) were predicted as having an active immune compartment, and the remaining samples that failed to be classified into these categories (with an FDR above 0.2) fell into the third cluster, termed the mixed type (Table S6). The GSEA of the hallmark gene sets (Table S5) showed that the presence of the active stroma subtype was associated with angiogenesis, EMT, and myogenesis (Figure 2C), and gene sets enriched in immune activation, such as the interferon alpha response, the gamma response and allograft rejection, were observed in the active immune subgroup (Figure 2D). The active immune subgroup exhibited a trend toward better recurrence-free survival than the other two subgroups (TCGA-COADREAD: cohort log-rank *P* = 0.036, Figure 2E; GSE39582 cohort: log-rank *P* = 0.021, Figure 2F), while no significant difference in overall survival was observed between these identified subtypes (data not shown).

*IFNG* produced by immune cells in the tumor environment plays an important role in recruiting *CD8* T cells and in NK cell infiltration (38). Cancers that feature high levels of angiogenesis factors usually exhibit an immunosuppressive phenotype, with high infiltration of regulatory T cells (Tregs), myeloid-derived suppressor cells (MDSCs), and tumor-associated macrophages (TAMs) (39). Overall, the active immune subtype exhibits favorable immune conditions against tumor initiation and progression, while the active stroma subtype exhibits adverse conditions.

### Demographic Characteristics

The distribution of sex, age at diagnosis and BMI were not different between these identified subtypes in TCGA-COADREAD cohort. Microsatellite instability-high (MSI-H) and -low (MSI-L) subtypes, CMS1 and CMS3, and early-stage tumors (stage I and stage II) were dominant in the active immune subtype, while the microsatellite stable (MSS) subtype, CMS4 and late-stage tumors (stage IV) were predominant in the active stroma subtype (Table 1).

**Table 1:** Distribution of clinical characteristics among TME subtypes.

**Variable**	**Active immune (192)**	**Active stroma (217)**	**Mixed type (235)**	***P*-test**
**Subtype MSI status (%)**				
MSI-H	24 (21.8)	4 (5.8)	13 (12.7)	0.028
MSI-L	15 (13.6)	9 (13.0)	22 (21.6)	
MSS	70 (63.6)	56 (81.2)	67 (65.7)	
Not evaluable	1 (0.9)	0 (0.0)	0 (0.0)	
**Sex (%)**				
Female	92 (47.9)	99 (45.8)	108 (46.4)	0.909
Male	100 (52.1)	117 (54.2)	125 (53.6)	
**Tumor stage (%)**				
Not reported	5 (2.6)	9 (4.2)	6 (2.6)	0.001
Stage I	50 (26.0)	30 (13.9)	31 (13.3)	
Stage II	73 (38.0)	66 (30.6)	98 (42.1)	
Stage III	47 (24.5)	71 (32.9)	65 (27.9)	
Stage IV	17 (8.9)	40 (18.5)	33 (14.2)	
**CMS subtype (%)**				
CMS1	53 (30.8)	13 (6.3)	39 (18.1)	<0.001
CMS2	51 (29.7)	60 (29.1)	81 (37.7)	
CMS3	56 (32.6)	14 (6.8)	26 (12.1)	
CMS4	12 (7.0)	119 (57.8)	69 (32.1)	
Age at diagnosis (median [IQR])	71.00 [62.00, 78.00]	67.00 [57.00, 74.25]	68.00 [59.00,77.50]	0.020 non-norm
BMI (median [IQR])	27.13 [23.87, 30.67]	27.10 [24.08, 30.99]	26.73 [23.63, 32.26]	0.901 non-norm

*Summary of the distribution of clinical characteristics among TME subtypes in the TCGA-COADREAD cohort. Numbers in the bracket placed immediately after the TME subtypes represent the absolute number of each group; numbers in other brackets represent the relative percentage distribution. For continuous variables, the median value with its interquartile range is shown. Fisher's test and the Kruskal-Wallis test were applied for categorical variables and continuous variables, respectively*.

### TME-Based Subtypes Related to the Immunotherapy Response

**Figure 3:**
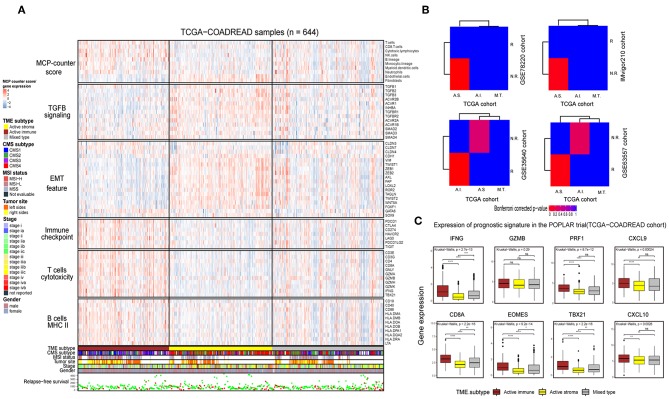
Identification of immune subtypes with distinct immune contexts and immunotherapeutic responses**. (A)** Complex heat map of the functional gene signature and immune cell infiltration score across immune subtypes in the TCGA-COADREAD cohort. The immune cell infiltration score was generated by MCP-counter. Corresponding feature names of the gene signatures are shown on the left side of the heat map. The TME subtype, CMS subtype, MSI status, tumor site, stage, sex and RFS are annotated in the lower panel. Color intensities indicate the expression level of the genes or the infiltration score of immune cells. **(B)** SubMap analysis of the TCGA-COADREAD cohort and four independent preimmunotherapeutic treatment datasets. The active stroma subtype shares high similarity with the immunotherapeutic resistance class in the GSE78220 and IMvigor210 cohorts, while the active immune subtype shares high similarity with the immunotherapeutic response class in the GSE35640 and GSE63557 cohorts, and the mixed type is not associated with either responders or non-responders. The colors labeled in each cell reflect the *P*-values of each subclass association. A.I., A.S., and M.T. represent the active immune, active stroma and mixed type, respectively. **(C)** Box plot of prognostic genes in the POPLAR study, with expression profiling among the immune classes. The gene expression level was normalized by log2 (TPM+1) transformation. The statistical significance of pairwise comparisons is annotated with symbols in which ns, *, **, and *** and **** represent not significant (*P* > 0.05), *P* ≤ 0.05, *P* ≤ 0.01, *P* ≤ 0.001, and *P* ≤ 0.0001, respectively. The Wilcoxon rank-sum test was used for comparisons between two groups, and the Kruskal–Wallis test was used for comparisons between more than two groups.

Immunotherapy can induce the durable remission of metastatic melanoma and non-small cell lung cancer (NSCLC), yet only a small subset of patients obtain a clinical response. Thus, detecting patients with high sensitivity to immunotherapy before implementing treatment is of vital importance. To comprehensively depict the immune landscape of the identified subtypes, published functional gene sets (Table S11) were adopted to perform immune context annotation. Unexpectedly, the active immune group showed enriched T cells, cytotoxic lymphocytes, a high expression of immune checkpoints (*PD1, PD-L1*, and *CTLA4*) and some active immune response factors, including T cell cytotoxicity factors (*GZMA, GZMB*, and *IFNG*), and B cell markers (*CD86* and *CD80*). In contrast, the active stroma group was enriched with endothelial cells, fibroblasts, EMT features and *TGF-*β signature genes (Figure 3A). Given the significant correlation between the expression of immune checkpoints (*PD-1, PD-L1*, and *CTLA-4*), the infiltration numbers of cytotoxic T cells and the immunosuppressive microenvironment with ICB responses (40, 41), we further explored the potential immunotherapy treatment benefit of the active immune group. SubMap analysis showed that the active stroma group shares high similarity with anti-*PD-1* resistance in melanoma patients (Figure 3B, top left) and metastatic urothelial tumors treated with *PD-L1* checkpoint inhibitors (Figure 3B, top right). The active immune class shares high similarity with melanoma patients who responded to treatment with *MAGE-A3* (Figure 3B, bottom left) and BALB/c mice who responded to treatment with anti-*CTLA-4* (Figure 3B, bottom right). Patients with a clinical response status to immunotherapy, including a complete response (CR) or partial response (PR), were considered immunotherapy responders, while those with stable disease (SD) or progressive disease (PD) were considered immunotherapy non-responders. Distinct immunotherapeutic regimens exhibited non-conformity with the identified immune subtypes, which might be due to the distinct resistance and reactive mechanisms of cancer cells adopted under different regimens and cancers (15, 42, 43). SubMap analysis on another colorectal cancer cohort, GSE39582 (Figure S3B), also achieved similar results, further confirming the non-conformity between the identified colorectal subtypes and the immunotherapy-treated cohorts that was mainly caused by distinct immunotherapy regimens and adopted tumor types. In addition, the expression of eight biomarkers established in the POPLAR trial (44), except for GZMB, was significantly higher in the active immune group than in the active stroma group (Figure 3C). By applying the identical analysis to the GSE39582 cohort, we obtained similar results (Figures S3A,B,D), which served as independent cross-validation. No difference was observed in tumor purity between these identified subtypes (Figure S3C, Table S9), thus eliminating potential bias elicited by differences in purity. Thus, our identified subtypes characterized by distinct immune contexts and their relationship with the immunotherapy treatment response may provide valuable information for clinical treatment decisions.

### Genomic Features of the TME-Based Subtypes

**Figure 4:**
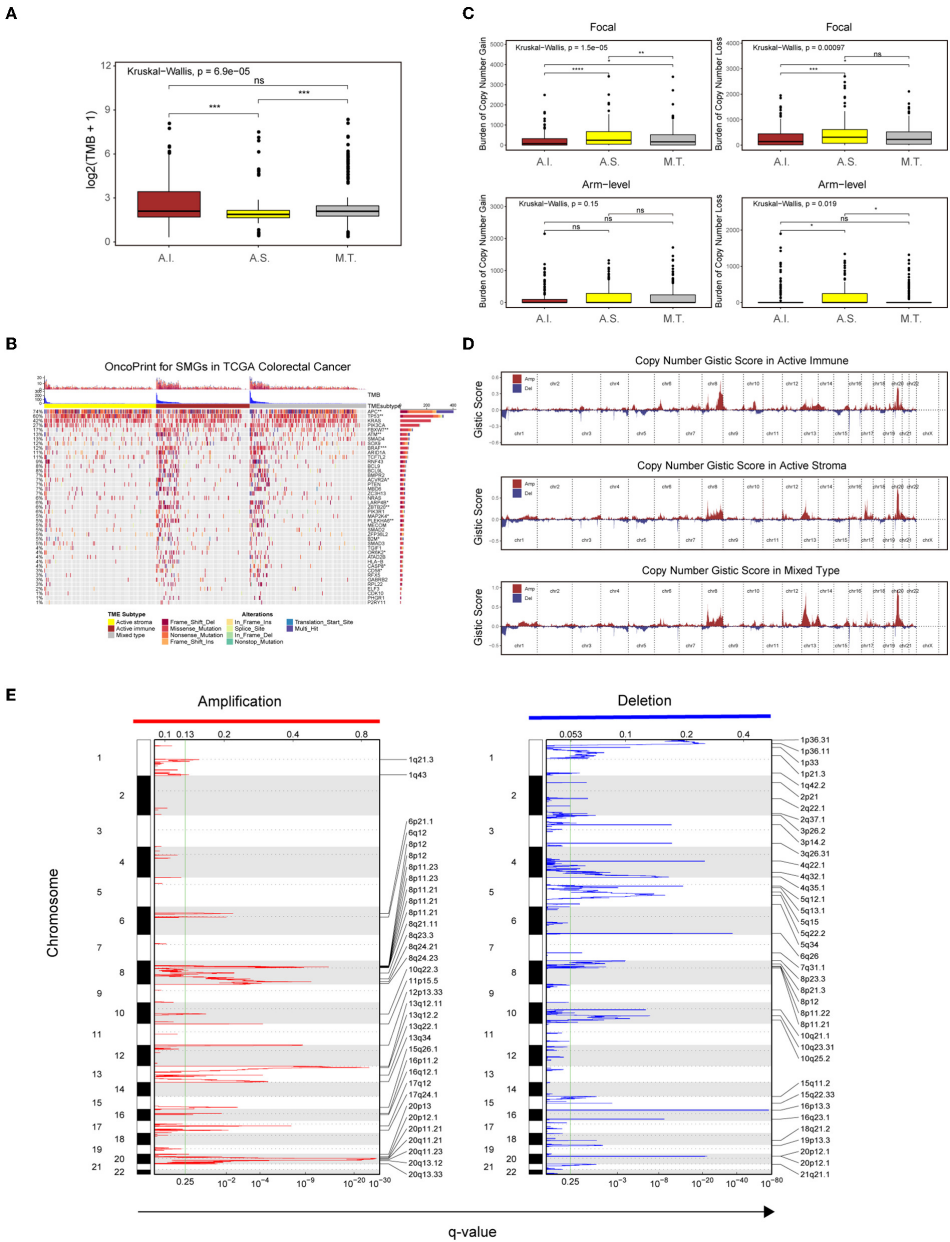
Genetic and copy number alterations across immune subtypes. **(A,C)** Distribution of TMB **(A)** and focal and broad copy number alterations **(C)** among the TME subtypes. The statistical significance of pairwise comparisons is annotated with symbols in which ns and *** represent not significant (*P* > 0.05) and *P* ≤ 0.001, respectively. A.I., A.S., and M.T. represent the active immune, active stroma and mixed type, respectively. **(B)** Mutation landscape of SMGs in the TCGA-COADREAD cohort. Genes are ordered by decreasing mutation frequency, and samples are sorted by the TMB in each subgroup. OncoPrint bar plot annotation; TMB and the TME subtype are annotated in the upper panel. Genes with a significant difference between the active immune and active stroma subtypes are annotated with symbols in which *, **, and *** represent *P* ≤ 0.05, *P* ≤ 0.01, and *P* ≤ 0.001, respectively. **(D)** Copy number profiles for the active stroma, active immune and mixed subtypes, with gains in dark red and losses in midnight blue. Gene segments are placed according to their location on chromosomes, ranging from chromosome 1 to chromosome 22. **(E)** Detailed cytoband with focal amplification (left) and focal deletion (right) in the active stroma group generated with GISTIC_2.0 software. The q value of each locus is plotted horizontally.

Recent analyses have linked the tumor genomic landscape with tumor cytolytic activity, indicating that a high TMB and specific somatic mutations are associated with antitumor immunity (45). The associated genomic data available in the TCGA database allowed us to investigate the underlying genomic mechanisms. In terms of TMB, patients in the active immune group and the mixed type group showed a higher TMB than those in the active stroma group (Table S10: active immune vs. active stroma: Wilcoxon rank-sum test *P* = 0.00016; mixed type vs. active stroma: Wilcoxon rank-sum test *P* = 0.00019). Notably, no significant difference was found between the active immune and mixed type groups (Table S10: active immune vs. mixed type: Wilcoxon rank-sum test *P* = 0.62), and the mixed type group did not exhibit particular immunologic characteristics or immunotherapeutic benefits compared with the active immune group (Figure 3B), which indicates that our immune subtype classification can identify patients with inflammatory milieu under similar TMB backgrounds. TMB of each sample in the TCGA-COADREAD cohort can be found in Table S8. To systematically elucidate the underlying genomic mechanism, we focused on 43 significantly mutated genes (SMGs) identified through MutSigCV_1.41 under a stringent threshold (*q* < 0.05) and identified 14 different mutated genes between the active stroma and active immune groups with a Benjamini-Hochberg-adjusted *P* < 0.05 (Figure 4B, Table S7). The identified mutated genes involved in antigen presentation (*B2M*) (45), cell cycle regulation (*FBXW7*) (46), the MAP kinase/ERK signaling pathway (*BRAF*) and the extrinsic apoptosis pathway (*CASP8*) (25)have been previously reported to be positively associated with immune cytolytic activity and the expression of costimulatory genes frequently mutated in the active immune group, while tumor suppressor genes including *TP53* and *APC* were more frequently mutated in the active stroma group. *TP53* (47) controls the expression of hundreds of genes involved in immunity, and the activation of the Wnt/beta-catenin pathway leads to a non-inflammatory milieu (48). These data suggest that tumor cells might adapt distinct escape mechanisms, primarily by impairing the extrinsic apoptosis pathway and antigen presentation in the active immune group and through the exclusion of immune effector cells in the active stroma group.

Given the recently reported evidence that a high burden of copy number loss is positively related to anti-CTLA-4 blockade resistance (49, 50), we next explored copy number alterations between these distinct immune groups. Similar to the findings of previous reports, patients within the active immune group showed a lower burden of gain and loss at the focal level and a lower burden of gain at the arm level compared with those in the active stroma group (Table S10: active immune vs. active stroma on focal-level gain burden: *P* = 0.0000023; focal-level loss burden: *P* = 0.00025; and arm-level gain burden: *P* = 0.053). Copy number burden of each sample in the TCGA-COADREAD cohort can be found in Table S8. Figure 4D shows the distribution of the G-score across all chromosomes in these subtypes. Focal amplifications (13q34, 20p11.21, and 20q13.33) and deletions (4q32.1, 5q15, and 5q34) within chromosomal regions were detected in the active stroma group (Figure 4E). The focal alterations of the active immune and mixed type groups are shown in Figure S4. The somatic copy number alteration (SCNA) level correlated with reduced cytotoxic immune infiltration, while the increased total mutation number correlated with high immune infiltration in colorectal cancer. The burden of copy number gain and loss in the mixed type group fell between those of the active immune and active stroma groups at the focal level (Figure 4C). It appears that focal copy number alterations strongly contribute to the difference in immune infiltration in colorectal cancer.

### Chemotherapeutic Treatment Response Tendency of the TME-Based Subtypes and Activation of the *NF-kB* Pathway in the Active Stroma Subtype

**Figure 5:**
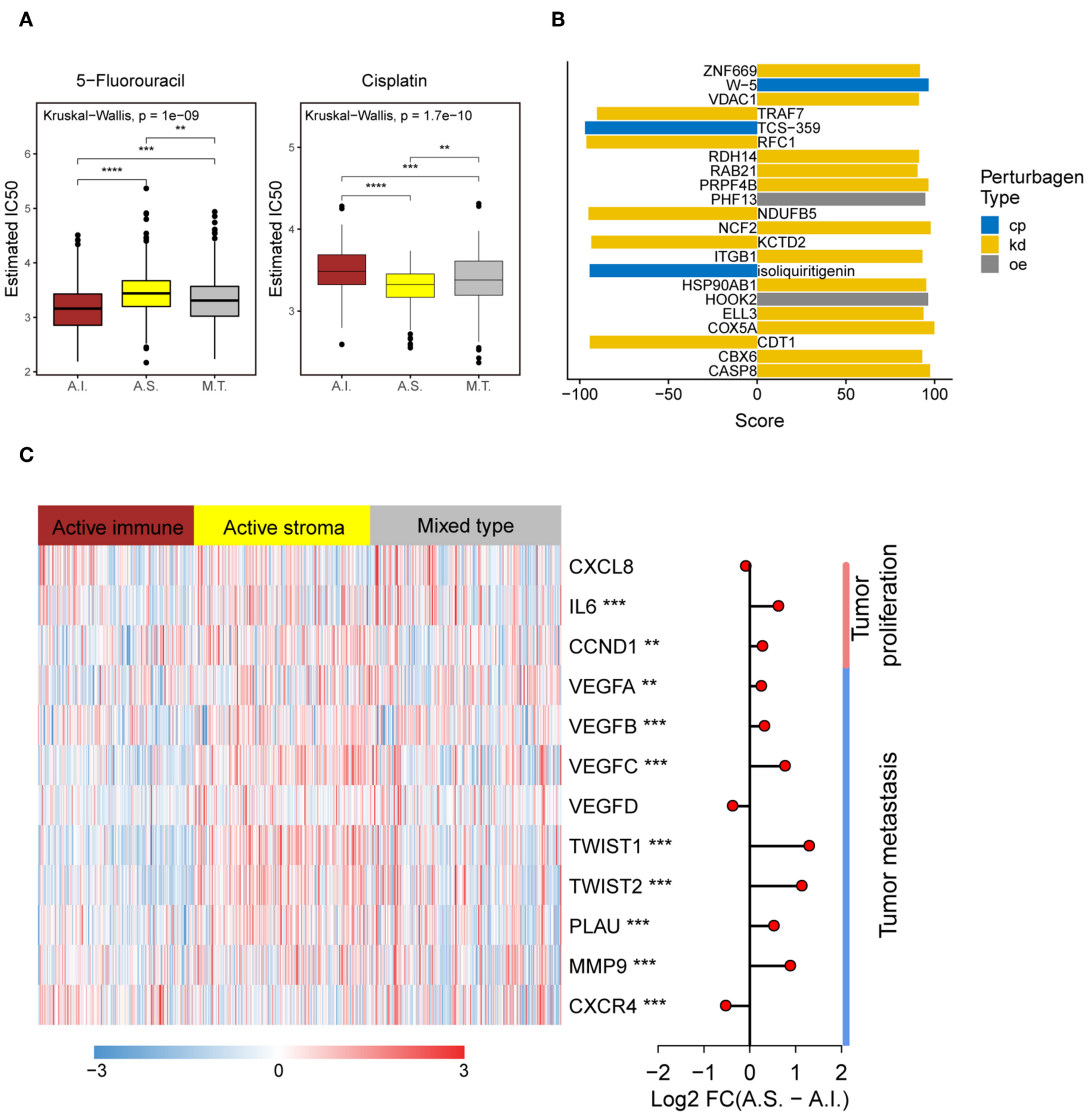
Different chemotherapeutic sensitivities across immune subtypes and activation of the NF-kB pathway in the active stroma. **(A)** Distribution of the estimated IC50 of 5-Fluorouracil and Cisplatin among the TME subtypes in TCGA-COADREAD cohort. The statistical significance of pairwise comparisons is annotated with symbols in which ns, *, **, and *** represent not significant (*P* > 0.05), *P* ≤ 0.05, *P* ≤ 0.001, and *P* ≤ 0.0001, respectively. A.I., A.S., and M.T. represent the active immune, active stroma and mixed type, respectively. **(B)** Bar plot of the candidate perturbations inferred from connectivity map analysis. Cp, kd, and oe represent compound, knockdown and overexpression, respectively. The score value placed on x-axis represents a holistic measurement of the relationship between the query gene set and the perturbation. The higher positive score, the more similar between the query and the perturbation. On the contrary, the lower negative score, the more opposing. **(C)** Complex heat map of the downstream genes of the *NF-kB* pathway. Genes with a significant difference between the active immune and active stroma subtypes are annotated with symbols in which **, and *** represent *P* ≤ 0.01, and *P* ≤ 0.001, respectively. Log2-fold change between the active stroma and active immune subtypes is shown on the right panel.

Platinum and 5-fluorouracil are widely used in the treatment of advanced colorectal cancer. Considering that chemotherapy is the most widely used strategy in the treatment of colorectal cancer, we used the “pRRophetic” package to predict the treatment response to 5-fluorouracil and cisplatin. The active immune subtype was more sensitive to 5-fluorouracil, while the active stroma subtype was more sensitive to cisplatin (Figure 5A) in TCGA-COADREAD cohort, strong concordance between chemotherapeutic treatment sensitive and TME-based subtypes was also seen in GSE39582 cohort (Figure S3F). This result can be used to guide the personalized treatment of colorectal cancer patients.

To identify candidate drugs and small molecules targeting the active stroma, we employed Connectivity Map (CMap) tools (34). CMap is a data-driven algorithm connecting genes, drugs and diseases and is widely used to discover potential therapeutic drugs and small molecules and to explore the mechanism of action underlying these drugs (51). We identified 22 candidate small molecules with absolute connectivity scores >90 (Figure 5B). We observed that knocking down the *TRAF7-*derived gene signature was strongly anticonnected with active stroma patients. *TRAF7* is a signal transducer for members of the TNF receptor superfamily, indicating the activation of the *NF-kB* pathway in the active stroma. It has been reported that the activation of *NF-kB* in pancreatic stellate cells prevents the infiltration of cytotoxic T cells by upregulating *CXCL12* in pancreatic cancer (52). Overexpression of its downstream genes, such as *MMP9* and *IL6*, was observed in the active stroma subtype compared with the active immune subtype (Figure 5C). Thus, strategies to deactivate the *NF-kB* pathway, such as blocking *TRAF7*, might be useful to enforce the infiltration of cytotoxic T cells and to kill colorectal cancer cells.

## Discussion

The colorectal gland epithelia and surrounding tumor environment interact and are mutually restricted during tumorigenesis. Normal colorectal glands mainly participate in digestion, iron metabolic processes and immunologic processes, including T cell activation, B cell activation and lymphocyte differentiation; these processes are enriched in its matching microenvironment to provide a defense mechanism against hostile factors to reach self-stabilization. In contrast, the tumor epithelium proliferates rapidly with the corresponding immunosuppressive environment, and thus, cancer cells are not expunged. Complementary changes in the tumor gland and microenvironment lead to cancer progression, metastasis and drug resistance. To date, no studies have systematically elaborated on the molecular interaction patterns of the epithelium and stroma during colorectal cancer initiation. Recently, rapidly developed technologies, such as single-cell RNA-seq, have allowed us to clarify the complex and dynamic relationship between cells (53, 54). However, the spatial location information of cells, which is of vital importance during tumorigenesis and progression, has been overlooked. Combining microdissection transcriptome profiling with single-cell RNA-seq or other developing technologies, such as Slide-seq (55), might help us determine spatial cell communication patterns.

We first revealed three molecular subsets, namely, the “active immune,” “active stroma” and “mixed type” subtypes based on the NTP method using gene templates established from stromal and immune compartments. The active immune and active stroma groups share similar characteristics with typically defined “hot tumors” and “cold tumors,” respectively. Hot tumors are characterized by a high degree of T cell and cytotoxic T cell infiltration and the overexpression of immune checkpoints such as PD-1, PD-L1, and LAG3 compared with cold tumors (56, 57). These identified subsets exhibited a distinct immune context, prognosis, and immunotherapy benefit, which supports the idea that the immune environment is of vital importance in predicting patient prognosis and evaluating the response rate of checkpoint inhibitor immunotherapies.

Genomic analysis revealed a distinct mutation and copy number change landscape. Patients in the active immune group exhibited a higher TMB, a lower copy number burden and enriched mutations that correlated with local immune cytolytic activity compared with those in the active stroma group. A significant negative correlation between TMB and the copy number alteration level has been observed in colorectal cancer (58). Ciriello et al. categorized colorectal cancer as an M class that was characterized by recurrent mutations other than recurrent copy number alterations, and McGrail et al. showed that neoantigen levels were predictive of cytotoxic T lymphocyte (CTL) infiltration (59, 60). However, Davoli et al. showed that arm/chromosome SCNAs provided a larger contribution to the immune signature than the total number of mutations, and the focal SCNA level failed to be selected for the prediction model. Immune cell infiltration mainly driven by mutations or copy number alterations is still a controversial issue. Our identified immune subtypes attached importance to focal number alterations. Nevertheless, these results revealed that distinct immunologic phenotypes have distinct genomic features (61).

Distinct immune cell infiltration patterns revealed a distinct tumor escape mechanism (62) and a remarkable difference in the prognosis of colorectal cancer (63).

The systematic analysis underscored the role of *NF-kB* pathway activation in leading low immune infiltration. Taken together, our results provide a systematic analysis of the biological changes in the epithelial and stromal compartments of colorectal cancer. Immunotherapy for colorectal cancer is approved only for microsatellite instability-high (MSI-H) patients, which comprise only a small subset (64), by the Food and Drug Administration (FDA). Since the TMB level of colorectal cancer ranks higher than that in other cancers (e.g., TCGA pan-cancer) (65), we have good reason to believe that the prospects for immunotherapy in colorectal cancer are optimistic. Strategies that block the *NF-kB* pathway might turn an active stroma compartment into an active immune compartment, thus increasing the opportunity for a response to ICB treatment and improving survival. The close interplay between intrinsic traits (mutation landscape and copy number alterations) and extrinsic characteristics (infiltrated immune cells) in our identified immune subtypes and their different tendencies toward the ICB treatment response might help guide immunotherapy decisions in colorectal cancer.

## Data Availability Statement

The datasets generated in this study can be found in the Gene Expression Omnibus (GEO) (https://www.ncbi.nlm.nih.gov/geo/) under the accession numbers GSE136735, and the access to other datasets used in this study can be found in the article when they are mentioned.

## Ethics Statement

The studies involving human participants were reviewed and approved by Ethics Committee of the National Cancer Center/Cancer Hospital, Chinese Academy of Medical Sciences and Peking Union Medical College. The patients/participants provided their written informed consent to participate in this study.

## Author Contributions

All authors have reviewed the data analyses. SC and LF conceived and designed the whole study. RS performed the data analyses and wrote the manuscript. BL collected clinical samples, and PL performed the microdissection and microarray experiments. BZ provided help with the computational analysis. All authors read, critically revised, and approved the final manuscript.

### Conflict of Interest

The authors declare that the research was conducted in the absence of any commercial or financial relationships that could be construed as a potential conflict of interest.

## Supplementary Material

Figure S1 Representative histopathological images of enrolled samples and the schematic diagram of manual microdissection. Representative histopathological image in hematoxylin-eosin staining of colorectal cancer tissue (A) in x100 and adjacent normal tissue (B) in 100x enrolled in this study (left) and the selected stromal compartment subjected to microdissection is noted in black circle (right).

Figure S2 Immune cell infiltration pattern in the epithelium and stroma of colorectal cancer and adjacent normal tissues and weighted gene coexpression network construction and evaluation. **(A)** Unsupervised hierarchical clustering analysis of the MCP-counter quantified immune scores in microdissection-processed colorectal cancer and adjacent normal tissues. The cluster distance was calculated with the Ward.D2 method. **(B)** Volcano plot showing immune cell enrichment differences between the normal stroma and tumor stroma. The Wilcoxon rank-sum test was used to compare differences, and the BH method was adopted to adjust *P*-values. **(C)** Scale-free fit index (left) and network mean connectivity (right) for various soft-thresholding powers. Red line in left plot indicates the predefined high value which is 0.9. Here, we choose the power 10 as the soft-thresholding power, which is the lowest power before the scale-free topology fit index curve reaching the predefined high value. **(D)** The scale-free plot indicates that our constructed network has a power-law degree distribution. **(E,F)** Network visualization of the selected gene template in the brown **(E)** and yellow **(F)** modules identified by WGCNA.

Figure S3 Identification of immune subtypes with distinct immune contexts and immunotherapeutic responses. **(A)** Heat map of the functional signature genes and immune cell infiltration extent across immune subtypes in the GSE39582 cohort. The immune cell infiltration score was generated by MCP-counter. The TME subtype, CMS subtype, MSI status, tumor site, stage, sex, and RFS are annotated in the lower panel. **(B)** SubMap analysis of the GSE39582 cohort and four independent preimmunotherapeutic treatment datasets. The active stroma subtype shares high similarity with the immunotherapeutic resistance class in the GSE78220 and IMvigor210 cohorts, while the active immune subtype shares high similarity with the immunotherapeutic response class in the GSE35640 and GSE63557 cohorts, and the mixed type is not associated with either responders or non-responders. The colors labeled in each cell reflect the *P*-values for each subclass association. A.I., A.S., and M.T. represent the active immune, active stroma and mixed type, respectively. **(C)** Distribution of tumor purity across immune subtypes in the TCGA-COADREAD (left) and GSE39582 (right) cohorts in which ns represents no significant difference been detected. **(D)** Box plot of prognostic genes in the POPLAR study, with expression profiling among the immune classes. The gene expression level was normalized by log2 (TPM+1) transformation. The statistical significance of pairwise comparisons is annotated with symbols in which ns, ^*^, ^**^, ^***^, and ^****^ represent not significant (*P* > 0.05), *P* ≤ 0.05, *P* ≤ 0.01, *P* ≤ 0.001, and *P* ≤ 0.0001, respectively. **(E)** Alluvial diagram showing associations among the TME subtype, CMS subtype and MSI status. **(F)** Distribution of the estimated IC50 of 5-Fluorouracil and Cisplatin among the TME subtypes in GSE39582 cohort. The statistical significance of pairwise comparisons is annotated with symbols in which ^*^, ^**^, and ^****^ represent *P* > 0.05, *P* ≤ 0.01, and *P* ≤ 0.0001, respectively. A.I., A.S., and M.T. represent the active immune, active stroma and mixed type, respectively. The Wilcoxon rank-sum test was used for comparisons between two groups, and the Kruskal–Wallis test was used for comparisons between more than two groups **(C,D,F)**.

Figure S4 Focal alterations in the active stroma and mixed type groups. **(A)** Detailed focal amplification (left) and focal deletion (right) in the active immune group generated with GISTIC_2.0 software. **(B)** Detailed focal amplification (left) and focal deletion (right) in the mixed type group generated with GISTIC_2.0 software.

Table S1 Clinical characteristics of enrolled samples in WGCNA analysis.

Table S2 Top 8000 genes with highest standard deviation in microdissection microarray.

Table S3 Gene Ontology-Biology process enrichment analysis of selected four module.

Table S4 Subtype template genes.

Table S5 Gene Set Enrichment Analysis of hallmark geneset derived from Molecular Signatures Database (MSigDB) in active immune and active stroma class.

Table S6 Nearest template prediction analysis on TCGA COAD-READ cohort and GSE39582 cohort.

Table S7 Wilcox test analysis on identified significant mutated genes between active stroma and active immune class.

Table S8 Tumor mutation burden and copy number burden among TME subtype.

Table S9 Tumor purity in TCGA COAD-READ cohort and GSE39582 cohort.

Table S10 Paired comparison detail among immune subtypes.

Table S11 Dataset and gene sets enrolled in this study.

## Abbreviations

ICB: immune checkpoint blockade
LCM: laser capture microdissection
TME: tumor microenvironment
AJCC: American Joint Committee on Cancer
TPM: transcripts per kilobase million
FPKM: fragments per kilobase million
CIBERSORT: cell type identification by estimating relative subset of known RNA transcripts
MCP-counter: Microenvironment Cell Populations-counter
PCA: principal component analysis
WGCNA: weighted gene coexpression network analysis
TMB: tumor mutation burden
CMS: consensus molecular subtype
NTP: nearest template prediction
TCGA: The Cancer Genome Atlas
SCNA: somatic copy number alteration
MSI-H: microsatellite instability-high
FDA, Food and Drug Administration. CMap: Connectivity Map.
