# Supplementary figures and images for: Identification of Distinct Immune Subtypes in Colorectal Cancer Based on the Stromal Compartment
Source: Front Oncol. 2020 Jan 10;9:1497. doi: 10.3389/fonc.2019.01497 (PMC6965328; doi:10.3389/fonc.2019.01497)

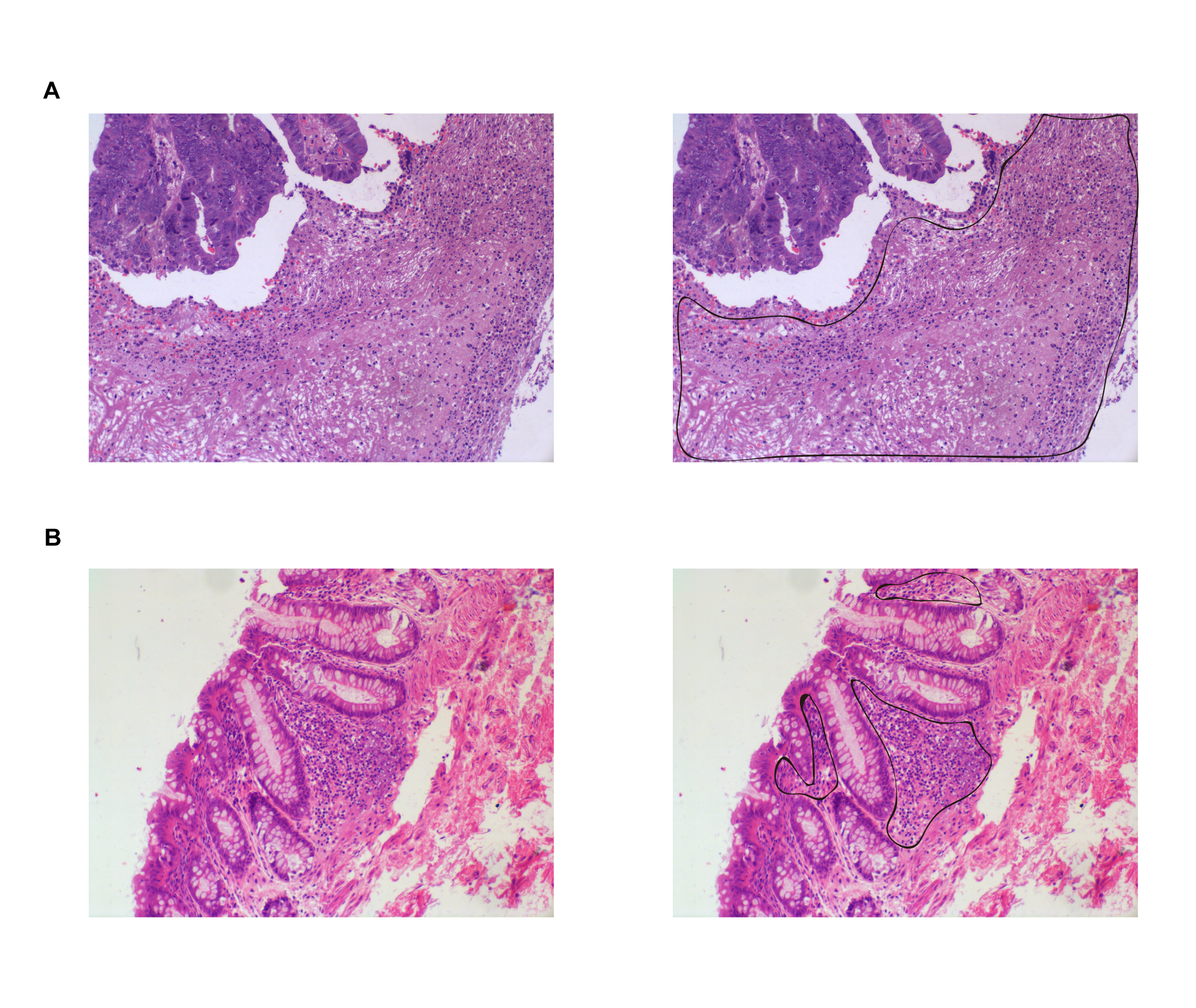

Supplement: Figure S1 — Representative histopathological images of enrolled samples and the schematic diagram of manual microdissection. Representative histopathological image in hematoxylin-eosin staining of colorectal cancer tissue (A) in x100 and adjacent normal tissue (B) in 100x enrolled in this study (left) and the selected stromal compartment subjected to microdissection is noted in black circle (right). [file Image_1.TIF]

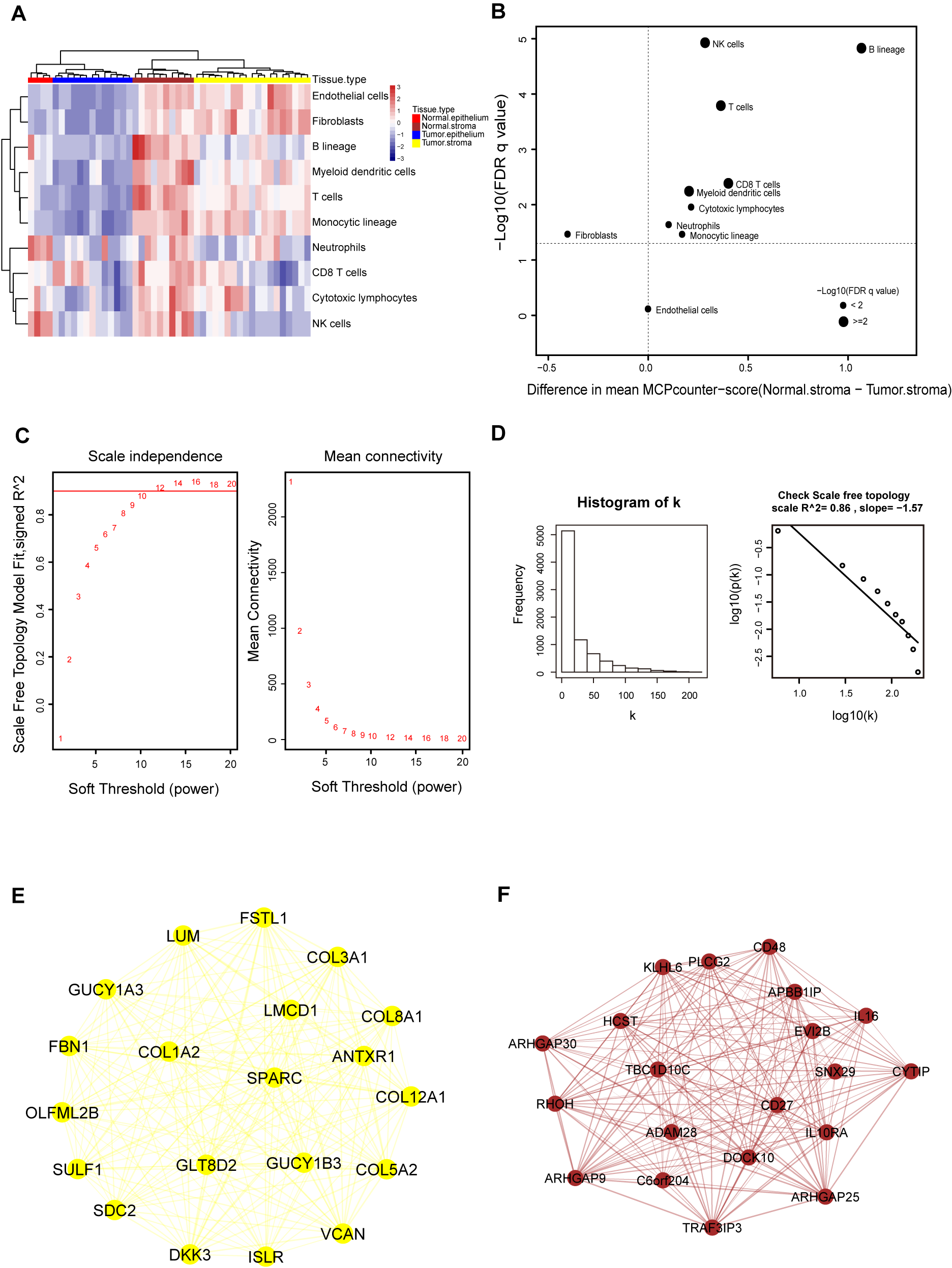

Supplement: Figure S2 — Immune cell infiltration pattern in the epithelium and stroma of colorectal cancer and adjacent normal tissues and weighted gene coexpression network construction and evaluation. (A) Unsupervised hierarchical clustering analysis of the MCP-counter quantified immune scores in microdissection-processed colorectal cancer and adjacent normal tissues. The cluster distance was calculated with the Ward.D2 method. (B) Volcano plot showing immune cell enrichment differences between the normal stroma and tumor stroma. The Wilcoxon rank-sum test was used to compare differences, and the BH method was adopted to adjust P-values. (C) Scale-free fit index (left) and network mean connectivity (right) for various soft-thresholding powers. Red line in left plot indicates the predefined high value which is 0.9. Here, we choose the power 10 as the soft-thresholding power, which is the lowest power before the scale-free topology fit index curve reaching the predefined high value. (D) The scale-free plot indicates that our constructed network has a power-law degree distribution. (E,F) Network visualization of the selected gene template in the brown (E) and yellow (F) modules identified by WGCNA. [file Image_2.TIF]

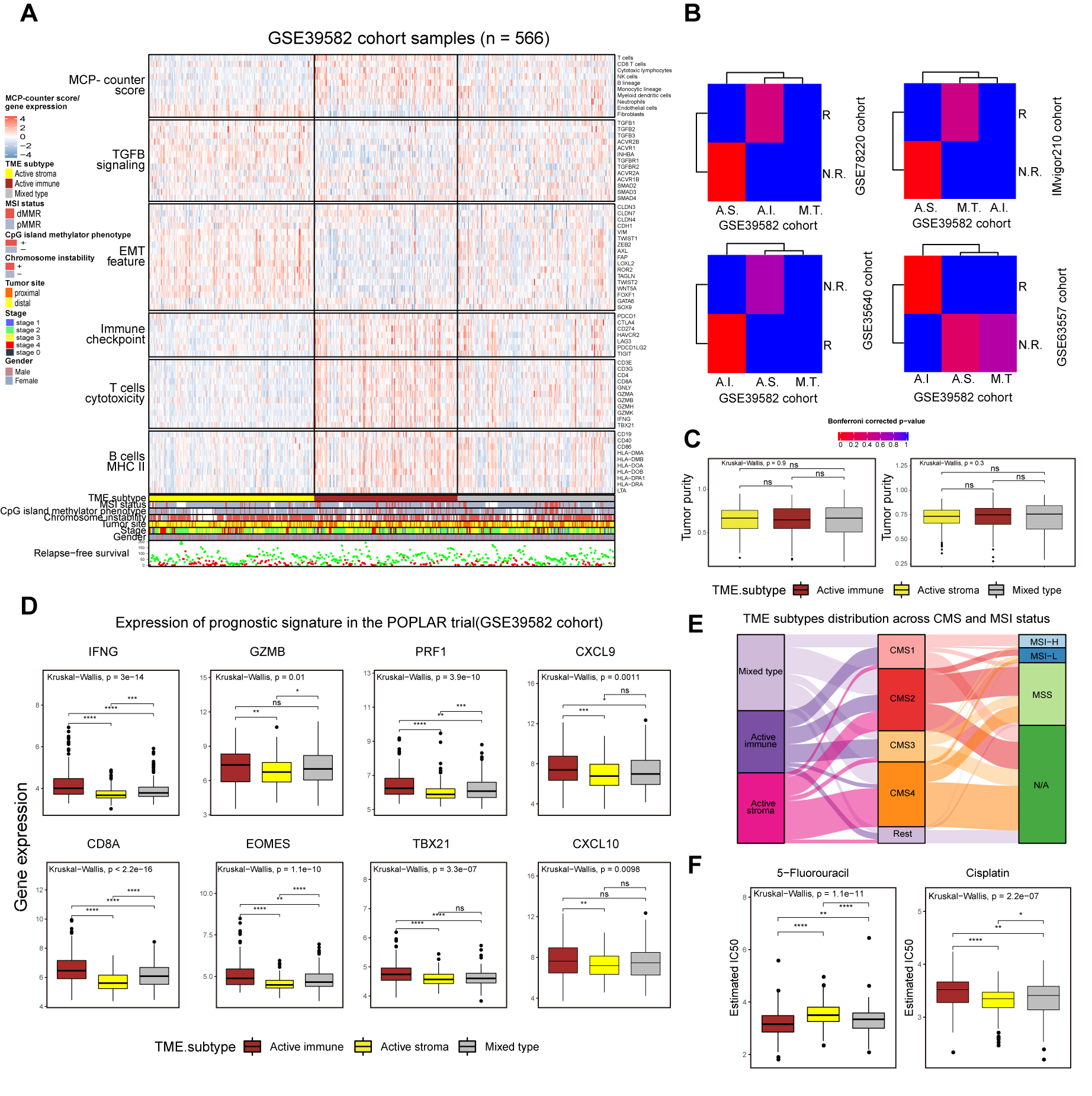

Supplement: Figure S3 — Identification of immune subtypes with distinct immune contexts and immunotherapeutic responses. (A) Heat map of the functional signature genes and immune cell infiltration extent across immune subtypes in the GSE39582 cohort. The immune cell infiltration score was generated by MCP-counter. The TME subtype, CMS subtype, MSI status, tumor site, stage, sex, and RFS are annotated in the lower panel. (B) SubMap analysis of the GSE39582 cohort and four independent preimmunotherapeutic treatment datasets. The active stroma subtype shares high similarity with the immunotherapeutic resistance class in the GSE78220 and IMvigor210 cohorts, while the active immune subtype shares high similarity with the immunotherapeutic response class in the GSE35640 and GSE63557 cohorts, and the mixed type is not associated with either responders or non-responders. The colors labeled in each cell reflect the P-values for each subclass association. A.I., A.S., and M.T. represent the active immune, active stroma and mixed type, respectively. (C) Distribution of tumor purity across immune subtypes in the TCGA-COADREAD (left) and GSE39582 (right) cohorts in which ns represents no significant difference been detected. (D) Box plot of prognostic genes in the POPLAR study, with expression profiling among the immune classes. The gene expression level was normalized by log2 (TPM+1) transformation. The statistical significance of pairwise comparisons is annotated with symbols in which ns, *, **, ***, and **** represent not significant (P > 0.05), P ≤ 0.05, P ≤ 0.01, P ≤ 0.001, and P ≤ 0.0001, respectively. (E) Alluvial diagram showing associations among the TME subtype, CMS subtype and MSI status. (F) Distribution of the estimated IC50 of 5-Fluorouracil and Cisplatin among the TME subtypes in GSE39582 cohort. The statistical significance of pairwise comparisons is annotated with symbols in which *, **, and **** represent P > 0.05, P ≤ 0.01, and P ≤ 0.0001, respectively. A.I., A.S., and M.T. represent [file Image_3.TIF]

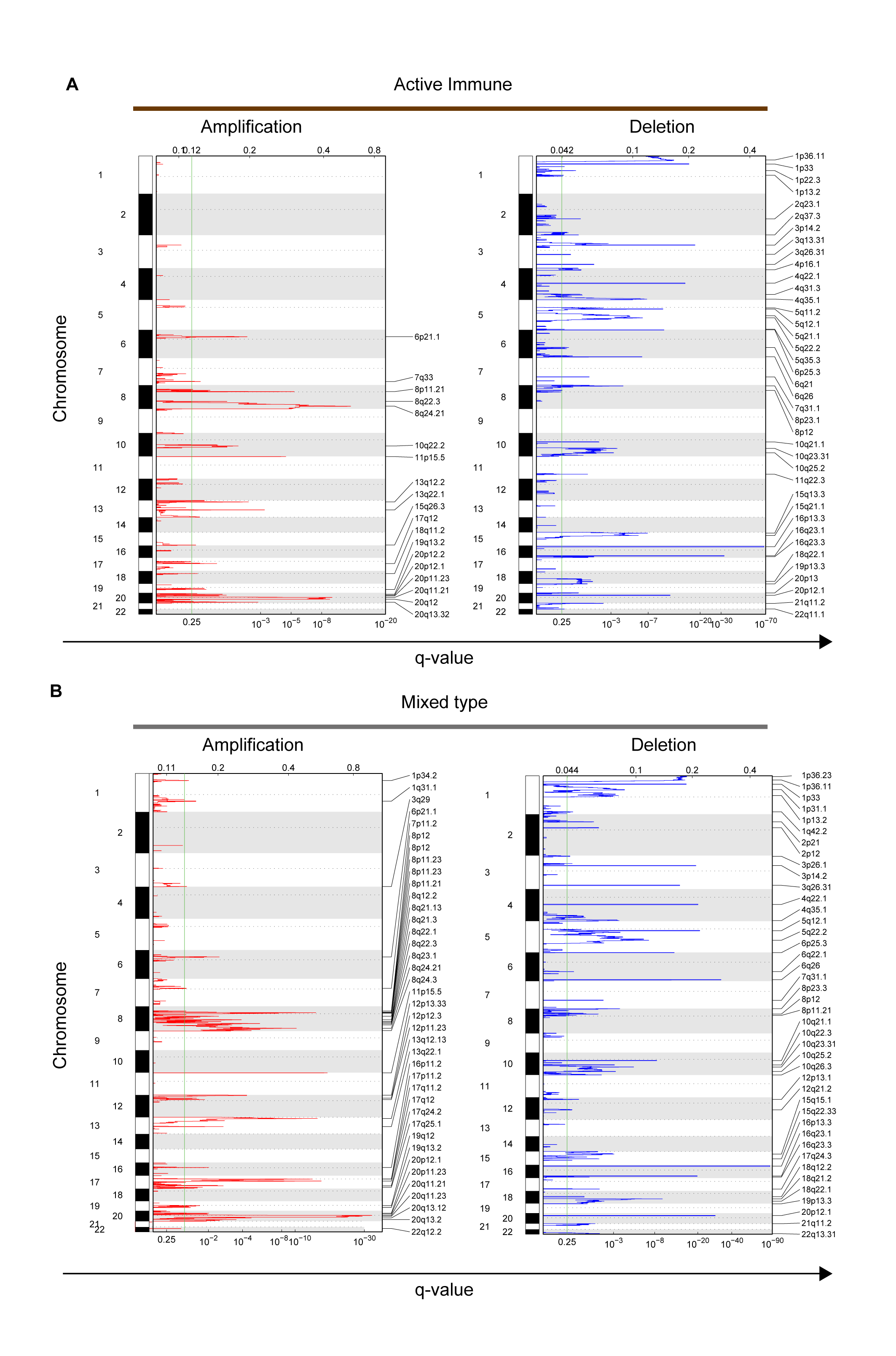

Supplement: Figure S4 — Focal alterations in the active stroma and mixed type groups. (A) Detailed focal amplification (left) and focal deletion (right) in the active immune group generated with GISTIC_2.0 software. (B) Detailed focal amplification (left) and focal deletion (right) in the mixed type group generated with GISTIC_2.0 software. [file Image_4.TIF]
